# Supplementary material for: Probing recombinant AAV capsid integrity and genome release after thermal stress by mass photometry
Source: Mol Ther Methods Clin Dev. 2024 Jun 24;32(3):101293. doi: 10.1016/j.omtm.2024.101293 (PMC11295964; doi:10.1016/j.omtm.2024.101293)
Supplement: Document S1. Figures S1–S6 and Table S1 [file mmc1.pdf]

OMTM, Volume *32*

Supplemental information

Probing recombinant AAV capsid integrity and genome release after thermal stress by mass photometry

Eduard H.T.M. Ebberink, Alisa Ruisinger, Markus Nuebel, Helena Meyer-Berg, Irene R.S. Ferreira, Marco Thomann, and Albert J.R. Heck

Table S1: Mass ranges used for quantification of AAV particles. Ranges are indicated for the different experiments in MDa.

	pBR322	***empty***	***partially filled***	***filled***	***overfilled***
no pBR322, no DNase					
AAV8_Rev_GFP	-	3.1-4.3	-	4.3-5.35	-
with pBR322					
AAV8_Rev_GFP	1.3-2.4	3.3-4.3	-	4.3-5.6	-
AAV8_Rev_empty	1.3-2.4	3.2-4.8	-	-	-
AAV8_Vir_GFP	1.3-2.5	3.3-4.1	-	4.1-4.85	4.85-5.6
AAV2_Rev_GFP	1.2-2.5	3.25-4.15	4.15-4.55	4.55-5.7	-
AAV9_FP	1.3-2.5	3.0-4.4	-	4.4-6.0	-
with DNase					
AAV8_Rev_GFP	-	3.1-4.3	-	4.3-5.35	-
AAV2_Rev_GFP	-	3.25-4.15	4.15-4.55	4.55-5.7	-
AAV8_Vir_GFP	-	3.3-4.05	-	4.05-4.8	4.8-5.6
AAV9_FP	-	3.1-4.4	-	4.4-6.0	-

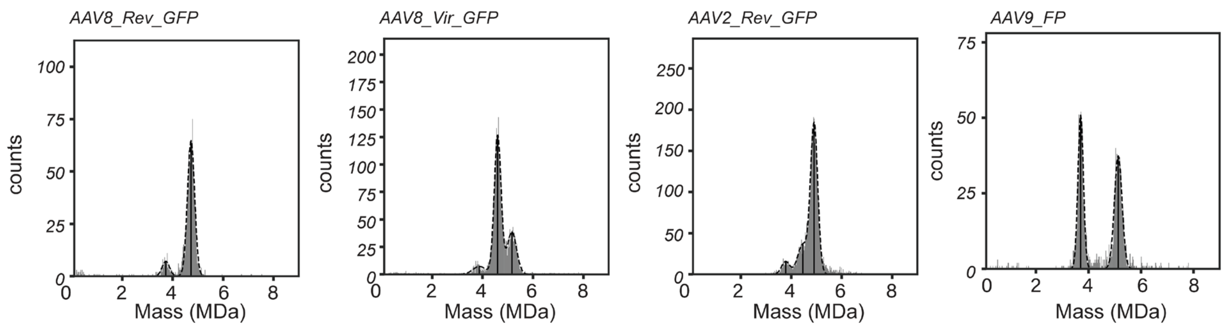

Figure S1: ***Mass photometry measurement of the different AAV preparations AAV8_Rev_GFP, AAV8_Vir_GFP, AAV2_Rev_GFP and AAV9_FP.*** Mass histograms derived from MP measurements on the AAV preparations that were assessed in this study prior to any treatments with heat or a nuclease. The MP measurements shown here were done at room temperature in PBS and on uncoated, glass slides. The AAV subpopulations were fitted with a Gaussian curve, indicated by black dashed lines. For every shown AAV preparation, there is a considerable amount of empty capsids present, especially in AAV9_FP. AAV8_Vir_GFP has capsids that contain more mass than expected based on a single genome (overfilled AAVs), while AAV2_Rev_GFP seems to have partially filled capsids.

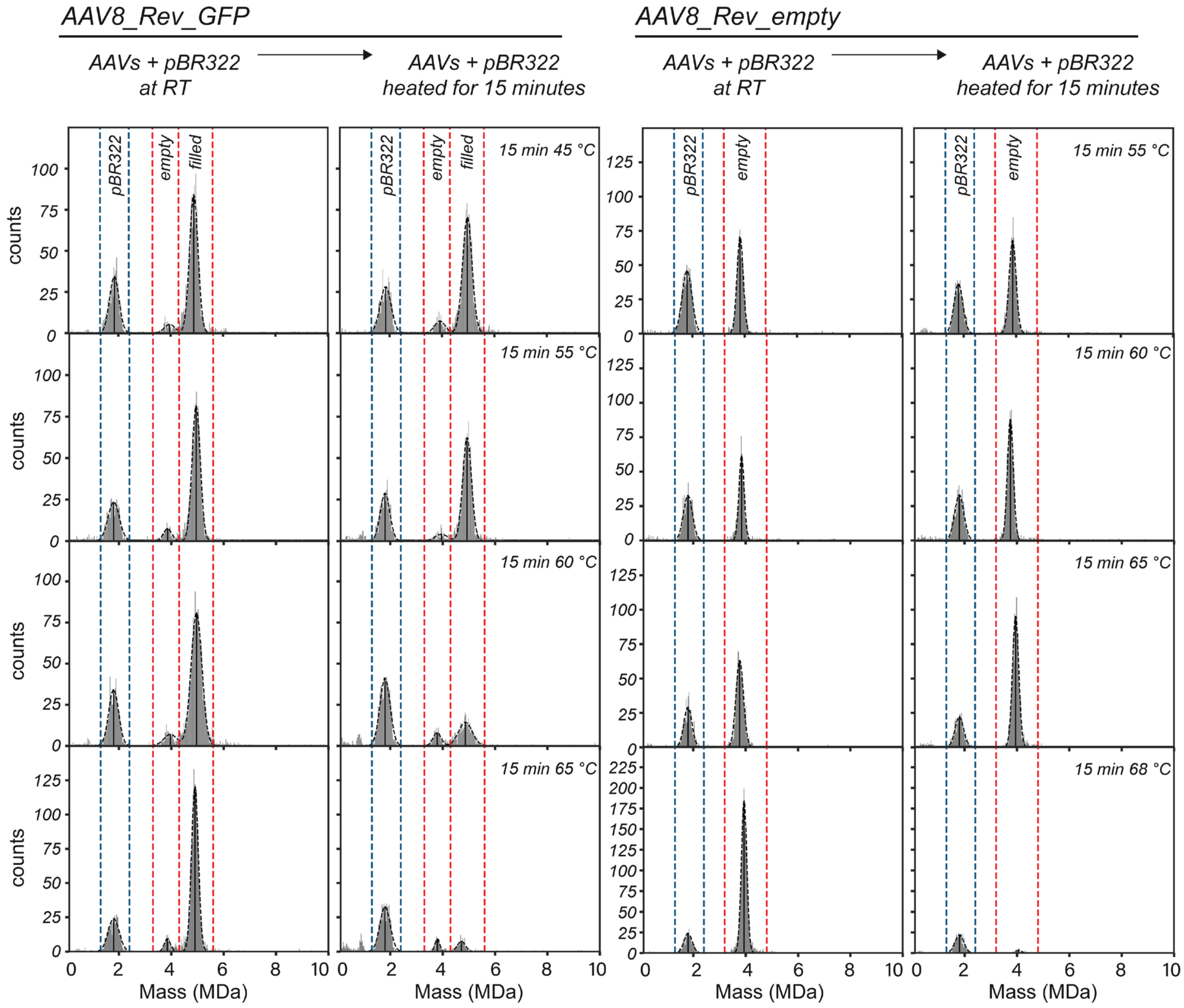

Figure S2: ***Mass photometry quantification before and after heat incubation with pBR322 of AAV8_Rev_GFP and AAV8_Rev_empty.*** Illustrative mass histograms derived from MP measurements of the AAV8_Rev preparations (either with a CMV-GFP transgene or empty) that were heat incubated together with the pBR322 DNA plasmid for quantification. A loss of AAVs can be seen when comparing the amounts of AAVs measured at room temperature (RT, left side) against those that were heated (right side). Gaussian fits of the AAVs and pBR322 are indicated by black dashed lines. The mass ranges that were used for quantification of the different AAV subpopulations (see Figure 3) are indicated by red dashed lines, the mass ranges for pBR322 quantification are indicated by blue dashed lines.

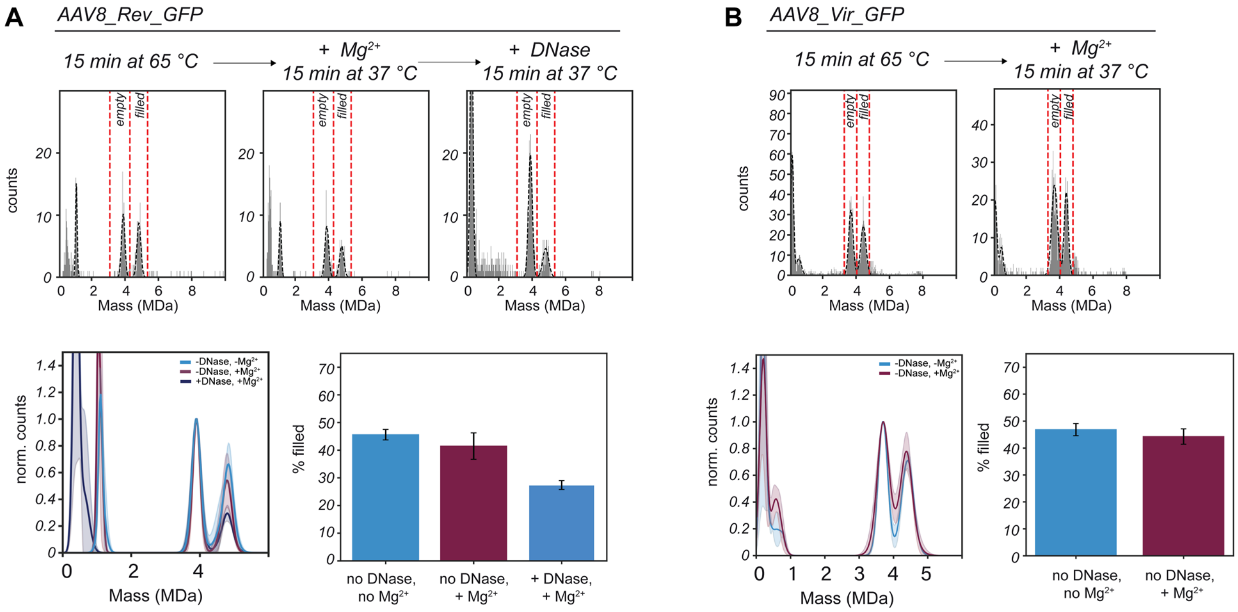

Figure S3: ***No effect of Mg^{2+} on the empty/filled distribution of AAV8 capsids.*** For both the AAV8 capsids (AAV8_Rev_GFP and AAV8_Vir_GFP), the change in empty and filled capsids was assessed by MP following the addition of Mg^{2+}. No effect could be detected of Mg^{2+} by itself. **A)** After heating AAV8_Rev_GFP at 65 °C for 15 minutes, the amount in empty and filled capsids was quantified within the specified range (indicated by red dashed lines). Three repeats were aligned and normalized on the most abundant AAV peak. The standard deviation is shown by semi-transparent bands. A bar plot is shown with the percentage of filled AAVs. Only after the addition of DNase, a major drop in filled particles can be seen. **B)** The same approach was made for AAV8_Vir_GFP, where also no difference could be seen for the addition of Mg^{2+} alone.

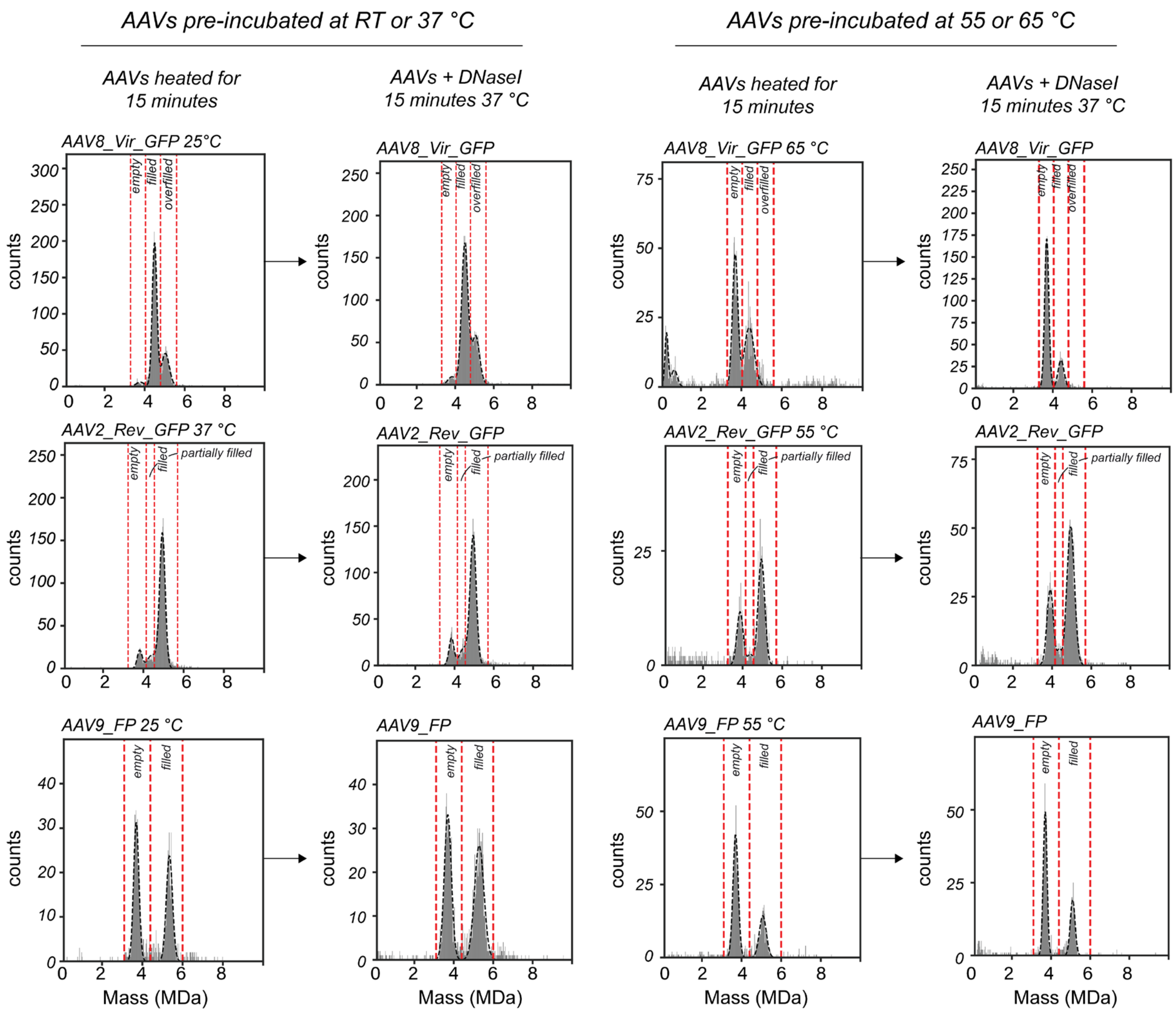

Figure S4: ***Mass photometry quantification of pre-heated AAV8_Vir_GFP, AAV2_Rev_GFP and AAV9_FP before and after treatment with DNase.*** Displayed are representative mass histograms of AAV8_Vir_GFP, AAV2_Rev_GFP, and AAV9_FP incubated at low or moderate temperatures (RT or 37 °C) or heated temperatures (55 or 65 °C) before and after the addition of DNase. The different AAV distributions were fitted with a Gaussian curve indicated by black dashed lines. Red dashed lines indicate the mass ranges used for quantifying the AAV particles (see Figure 5).

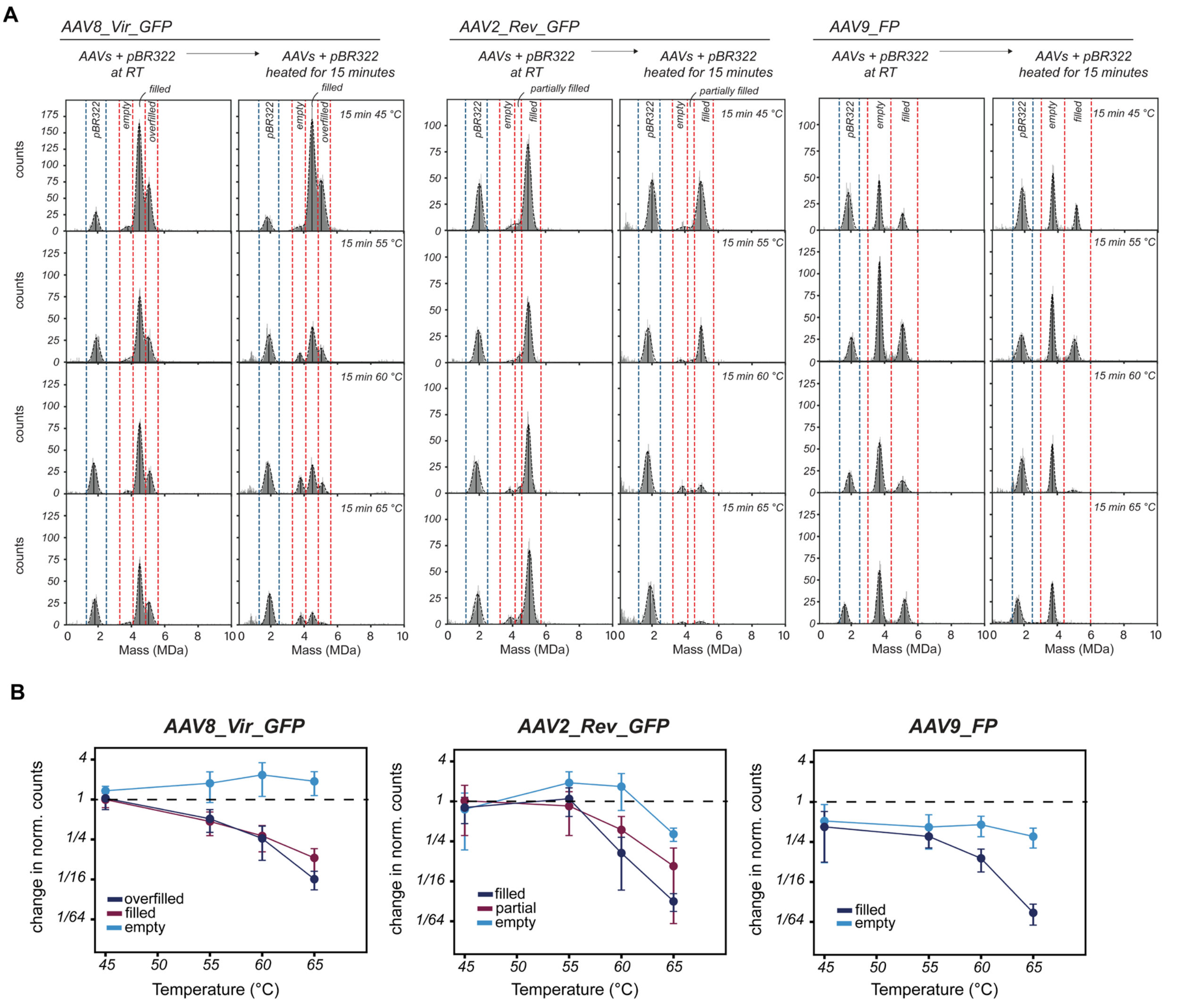

Figure S5: ***Mass photometry quantification of thermal-stressed AAV8_Vir_GFP, AAV2_Rev_GFP and AAV9_FP together with the pBR322 plasmid standard.*** **A)** Illustrative mass histograms derived from MP measurements of the heat incubated AAV8_Vir_GFP, AAV2_Rev_GFP and AAV9_FP. AAVs were incubated at 45, 55, 60 and 65 °C together with the pBR322 reference plasmid for quantification and comparison to incubation at room temperature (RT). Gaussian fits of the AAVs and pBR322 are indicated by a black dashed line. The mass ranges that were used for quantification of the different AAV subpopulations are drawn by red dashed lines, the mass range used for pBR322 quantification is drawn by blue dashed lines. **B)** The heat induced loss of AAV8_Vir_GFP, AAV2_Rev_GFP and AAV9_FP was derived following normalization of the pBR322 signal before and after heating. Displayed are the relative changes in AAV subpopulations following incubation at room temperature compared to incubation at 45, 55, 60 and 65 °C. Error bars represent the standard deviation between the different repeats.

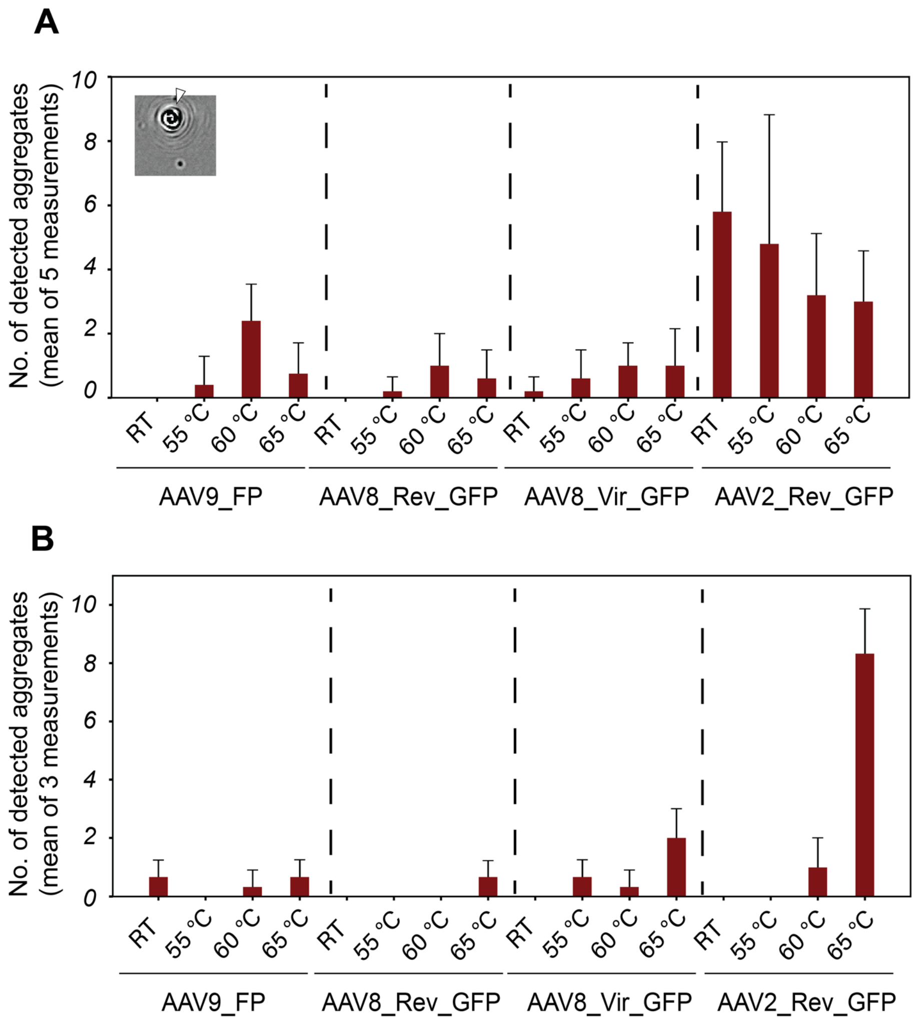

Figure S6: ***Aggregation of AAVs following heating.*** Aggregates were identified by visual inspection of the MP recordings after incubation of 15 minutes at the indicated temperatures. **A)** Recordings of experiments from Figure 3 and S5 that were performed on APTES slides were analyzed for aggregates. Within the AAV2_Rev_GFP sample, considerably more aggregates could be seen than in the other AAVs. Inset displays an example of an aggregate landing event. **B)** Recordings of experiments from Figures 4 and 5 performed on glass slides were analyzed for aggregates. Here, AAV2_Rev_GFP showed prominent aggregation after heating at 65 °C. Error bars in both panels represent the standard deviation between the different repeats.
